# Drug Conjugates Based on a Monovalent Affibody Targeting Vector Can Efficiently Eradicate HER2 Positive Human Tumors in an Experimental Mouse Model

**DOI:** 10.3390/cancers13010085

**Published:** 2020-12-30

**Authors:** Tianqi Xu, Haozhong Ding, Anzhelika Vorobyeva, Maryam Oroujeni, Anna Orlova, Vladimir Tolmachev, Torbjörn Gräslund

**Affiliations:** 1 Department of Immunology, Genetics and Pathology, Uppsala University, 751 85 Uppsala, Sweden; tianqi.xu@igp.uu.se (T.X.); anzhelika.vorobyeva@igp.uu.se (A.V.); maryam.oroujeni@igp.uu.se (M.O.); vladimir.tolmachev@igp.uu.se (V.T.); 2 Department of Protein Science, KTH Royal Institute of Technology, Roslagstullsbacken 21, 114 17 Stockholm, Sweden; haozhong@kth.se; 3 Research Centrum for Oncotheranostics, Research School of Chemistry and Applied Biomedical Sciences, Tomsk Polytechnic University, 634050 Tomsk, Russia; anna.orlova@ilk.uu.se; 4 Department of Medicinal Chemistry, Uppsala University, 751 23 Uppsala, Sweden

**Keywords:** affibody molecule, human epidermal growth factor receptor 2, HER2, SKOV3, DM1, albumin binding domain

## Abstract

**Simple Summary:**

Drug conjugates, consisting of a tumor targeting part coupled to a highly toxic molecule, are promising for treatment of many different types of cancer. However, for many patients it is not curative, and investigation of alternative or complimentary types of drug conjugates is motivated. Here, we have devised and studied a novel cancer cell-directed drug conjugate Z_HER2:2891_-ABD-E_3_-mcDM1. We found that it could induce efficient shrinkage and, in some cases, complete regression of human tumors implanted in mice, and thus holds promise to become a therapeutic agent for clinical use in the future.

**Abstract:**

The human epidermal growth factor receptor 2 (HER2) is frequently overexpressed in a variety of cancers and therapies targeting HER2 are routinely used in the clinic. Recently, small engineered scaffold proteins, such as affibody molecules, have shown promise as carriers of cytotoxic drugs, and these drug conjugates may become complements or alternatives to the current HER2-targeting therapies. Here, we investigated if a monovalent HER2-binding affibody molecule, Z_HER2:2891_, fused with a plasma half-life extending albumin binding domain (ABD), may be used as carrier of the cytotoxic maytansine derivate mcDM1. We found that the resulting drug conjugate, Z_HER2:2891_-ABD-E_3_-mcDM1, had strong affinity for its cognate molecular targets: HER2 and serum albumin. Z_HER2:2891_-ABD-E_3_-mcDM1 displayed potent cytotoxic activity towards cells with high HER2 expression, with IC_50_ values ranging from 0.6 to 33 nM. In vivo, an unspecific increase in uptake in the liver, imparted by the hydrophobic mcDM1, was counteracted by incorporation of hydrophilic and negatively charged glutamate residues near the site of mcDM1 conjugation. A dose-escalation experiment showed that increasing doses up to 15.1 mg/kg gave a proportional increase in uptake in xenografted HER2-overexpressing SKOV3 tumors, after which the tumors became saturated. Experimental therapy with four once-weekly injection of 10.3 or 15.1 mg/kg led to efficient regression of tumors in all animals and complete regression in some. Weight loss was detected for some animals in the group receiving the highest dose, suggesting that it was close to the maximum tolerated dose. In conclusion, the monovalent HER2-targeting affibody drug conjugate presented herein have potent anti-tumor activity in vivo.

## 1. Introduction

An emerging trend in recent years has been the development of targeted drug conjugates, where a targeting domain is coupled to a cytotoxic drug, allowing for selective killing of cancer cells. The most common format consists of a monoclonal antibody (mAb), decorated with multiple copies of a drug, a so-called antibody drug conjugate (ADC) [1].

The human epidermal growth factor receptor 2 (HER2) is overexpressed in a sub-set of patients with e.g., breast, ovarian, and gastric cancer, and has limited expression on normal cells. Several drugs selectively targeting HER2 have been described and one of the first ADCs approved for clinical use by the US Food and Drug Administration, was trastuzumab emtansine (T-DM1), targeting HER2. It is approved for therapy of metastatic HER2-positive breast cancer, and has been found to significantly increase progression-free survival among patients compared to standard treatment [2]. However, most patients treated with T-DM1 eventually progress [2,3], which motivates investigation and development of alternative or complimentary treatment options including novel drugs.

An important property of targeted drug conjugates is their ability to efficiently penetrate solid tumor mass to deliver a sufficiently high concentration of the cytotoxic payload to all neoplastic cells. In a pre-clinical mouse model of breast cancer, it was found that smaller JIMT-1 tumors (approximately 70 mm^3^) were more sensitive to treatment with T-DM1 than larger tumors (approximately 350 mm^3^) [4]. One reason for the poorer response of the larger tumors could be inefficient tumor penetration. Due to insufficient lymphatic drainage, the rate of tumor penetration is typically diffusion dependent, and the relatively large size of a mAb hampers its diffusivity. The use of smaller proteins and peptides as targeting vehicles could be advantageous as their rate of diffusion is higher [5,6,7,8].

Moreover, the large size and complex structure of mAbs makes site-specific conjugation of the drug and control of the drug-to-antibody ratio difficult, which may affect the binding properties [9] and the in vivo stability of the resulting ADC [10]. Even though several strategies for site-specific conjugation have been described, many ADCs in clinical development still rely on non-site-specific conjugation of the drug to lysines or cysteines [11].

Engineered scaffold proteins (ESPs) may be used as an alternative to mAbs for targeting tumor cells and have recently been investigated as carriers of cytotoxic drugs [12,13]. Affibody molecules constitute a class of ESPs, consisting of 58 amino acids arranged in an anti-parallel three-helix bundle [14]. The use of affibody molecules for specific delivery of cytotoxic drugs to cancer cells may provide several advantages compared to mAbs. First, affibody molecules are much smaller (approx. 7 kDa), which ensures rapid extravasation into the extracellular space, as well as more efficient penetration of poorly perfused solid tumors. Second, since the affibody scaffold does not contain cysteines, one or more cysteines may be introduced at desired positions using genetic engineering techniques, which may be utilized for site-specific conjugation of cytotoxic drugs using thiol-directed chemistry. Clinical testing of affibody molecules targeting different cell surface receptors have shown that they are well tolerated by patients without causing immune reactions. For example, radionuclide molecular imaging of HER2-expressing primary tumors and metastases by PET and SPECT successfully met the trial’s endpoints without causing adverse effects [15,16], validating the clinical suitability of the affibody class of ESPs. In addition, repeated administration to rats of a HER2-targeting affibody tracer for molecular imaging showed that no anti-affibody antibodies were formed [17]. Together, these features make affibody molecules promising carriers for tumor-targeted drug delivery.

The small size of affibody molecules provides fast clearance from blood through filtration in the kidneys. A strategy previously explored to extend the plasma half-life of affibody molecules was by fusion with an albumin binding domain (ABD) [18]. The ABD binds with high affinity to serum albumin (SA) in blood, which increases the molecular weight by 67 kDa of the affibody-ABD/SA complex to above the cut-off of kidney filtration [19].

Previously, anti-HER2 affibody-drug conjugates (AffiDCs) containing an ABD and the cytotoxic drug mcDM1 have been investigated [13]. The AffiDCs demonstrated prolonged retention in circulation compared to HER2-binding affibody molecules alone, and increased survival of mice bearing HER2-expressing SKOV3 xenografts. It was found that the introduction of mcDM1 caused an increase in hydrophobicity, which resulted in an increase in hepatic uptake in vivo. Minimizing hepatic uptake is important because drug-induced liver injury (DILI) is a common cause of drug withdrawal from the market [20], and is sometimes a difficulty also with ADCs [11]. An effort to reduce the hepatic uptake of the HER2-targeting AffiDC was undertaken by introducing three or six glutamic acid residues next to the cysteine carrying mcDM1 [21]. The resulting conjugates retained high tumor uptake while the unspecific uptake in liver was significantly reduced.

An essential aspect of molecular design is the binding valency of the cell targeting module. Previous studies on HER3-targeting, employing ABD-fused affibody molecules, have revealed that a bivalent format increases the rate of internalization [22]. A high rate of internalization of AffiDCs is desirable since internalization is required for drug action. However, a bivalent format may not be advantageous for HER2-targeting affibody molecules armed with mcDM1, since mono- and divalent constructs have been found to have a similar cytotoxic potency in vitro [13].

The cytotoxic payload mcDM1 is derived from the cytotoxic compound maytansine and acts as a microtubule polymerization inhibitor that disrupts the microtubule network, causing cell death [23]. The maleimidocaproyl (mc) linker is non-cleavable and drug conjugates equipped with it first engage their intended target on the cell surface, after which the conjugate is internalized through receptor-mediated endocytosis. It is then transported to the lysosomes where the protein part is degraded, leading to release of mcDM1, which diffuses to the cytosol where the poisoning process occurs.

**Figure 1:**
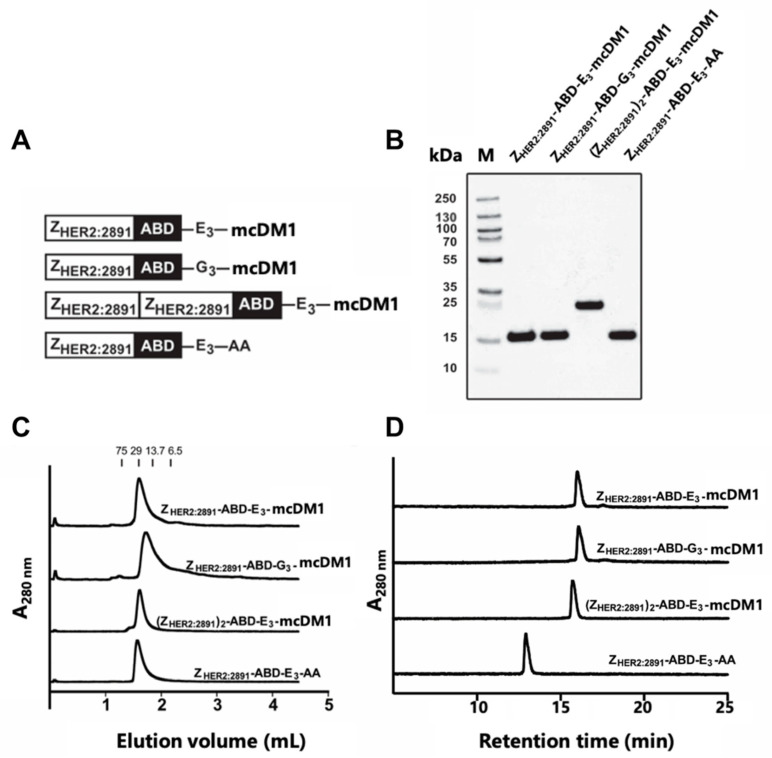
Biochemical characterization of the affibody-drug conjugates (AffiDCs). (**A**) Schematic description of the AffiDCs and the non-toxic control Z_HER2:2891_-ABD-E_3_-AA. A G_4_S linker was used to connect Z_HER2:2891_ domains and the albumin binding domain (ABD) in all constructs. (**B**) SDS-PAGE analysis under reducing conditions. The numbers to the left correspond to the molecular weight in kDa of marker proteins in lane M. (**C**) Size-exclusion chromatography analysis. The numbers above the chromatograms corresponds to the elution volumes of protein standards with the indicated molecular weights (kDa). (**D**) RP-HPLC analysis during a 20 min linear gradient from 30% to 60% acetonitrile in water supplemented with 0.1% TFA.

In this study, a monovalent HER2-binding affibody drug conjugate, Z_HER2:2891_-ABD-E_3_-mcDM1, was investigated, consisting of a HER2 binding affibody molecule (Z_HER2:2891_) coupled to an ABD for in vivo half-life extension and a tri-glutamyl (E_3_) linker to minimize hepatic uptake, conjugated with the maytansine derivate mcDM1. The properties of monovalent Z_HER2:2891_-ABD-E_3_-mcDM1 was compared to the properties of divalent (Z_HER2:2891_)_2_-ABD-E_3_-mcDM1 [21] to investigate the impact of targeting protein valency. As control of the impact of the tri-glutamyl linker, a construct where the E_3_-linker was replaced with an uncharged and less hydrophilic tri-glycine linker (G_3_), was created (Z_HER2:2891_-ABD-G_3_-mcDM1). A non-toxic control, Z_HER2:2891_-ABD-E_3_-AA with the C-terminal cysteine capped by reaction with iodoacetamide was also included. The four constructs are schematically represented in Figure 1A. After thorough in vitro characterization, we investigated biodistribution and potency for targeted therapy in a xenograft model of HER2-positive cancer in vivo.

## 2. Results

### 2.1. Design, Production and Biochemical Characterization of AffiDCs

The affibody fusion proteins were recombinantly expressed and purified, and mcDM1 was conjugated to a unique cysteine, placed in the C-terminus. After further purification, the conjugates were analyzed by SDS-PAGE under reducing conditions, and the gel showed pure conjugates with essentially the expected molecular weights (Figure 1B). The conjugates were further analyzed by size-exclusion chromatography under native conditions and were eluted as single peaks, showing no degradation or formation of aggregates (Figure 1C). The conjugates were also analyzed by passage through a C18 column in a RP-HPLC setup, where elution was carried out by a linear gradient of acetonitrile in water (Figure 1D). The area under curves in the recorded chromatograms showed that all conjugates had a purity of >95% (Table 1).

The non-toxic control, Z_HER2:2891_-ABD-E_3_-AA, lacking mcDM1, was eluted earlier than the other three, suggesting an increase in hydrophobicity of the conjugates by coupling to mcDM1. The molecular weights were measured by mass spectrometry and the results showed conjugates matching exactly the theoretical molecular weight with a drug-to-affibody ratio of 1 (Table 1, Figure S1). A non-target control Z_Taq_-ABD-mcDM1 was also produced and was likewise found to be of high purity and with a correct molecular weight (Figure S2). This conjugate interacts specifically with Taq DNA polymerase, and thus not with any protein of mammalian origin.

### 2.2. Binding Specificity and Affinity Determination

To investigate if mcDM1 conjugation or linker composition, E_3_ or G_3_, next to the cysteine where mcDM1 was attached, affected the affinity of Z_HER2:2891_ to HER2, dilution series of the conjugates were injected into a biosensor over a surface with immobilized HER2 followed by determination of the kinetic parameters (Figure S2, Table 2).

The dissociation rates (k_d_) were found to be similar for the three monovalent affibody conjugates and ranged from 2.2 to 2.4 × 10^−4^ s^−1^. The association rates for the two monovalent AffiDCs, Z_HER2:2891_-ABD-E_3_-mcDM1 and Z_HER2:2891_-ABD-G_3_-mcDM1, were slower (2.9 × 10^5^ M^−1^·s^−1^ and 4.0 × 10^5^ M^−1^·s^−1^, respectively) compared with the non-toxic control Z_HER2:2891_-ABD-E_3_-AA (8.09 × 10^5^ M^−1^·s^−1^). Unlike the monovalent AffiDCs, the dimeric (Z_HER2:2891_)_2_-ABD-E_3_-mcDM1 has two domains which may interact with HER2. Its kinetic parameters were therefore not readily comparable with that of the monomeric constructs, but by visual inspection it was evident that the dissociation rate is slower, suggesting a cooperativity of the domains in the interaction with the HER2-surface. The equilibrium dissociation constants (K_D_) for the interactions were calculated and are displayed in Table 2.

The ability of the conjugates to interact with human serum albumin (HSA) and mouse serum albumin (MSA) was investigated by injection of dilution series over a chip with immobilized HSA or MSA (Figure S3). The affinities (K_D_) of the monomeric constructs were derived from the sensorgrams and are displayed in Table 2. Due to faster dissociation, the affinities between the monovalent AffiDCs and MSA were slightly weaker than the affinities for HSA. For the dimeric (Z_HER2:2891_)_2_-ABD-E_3_-mcDM1, the affinity was weaker to both HSA and MSA compared to the monomers as a result of slower on-rates.

### 2.3. In Vitro Cytotoxicity Analysis

**Figure 2:**
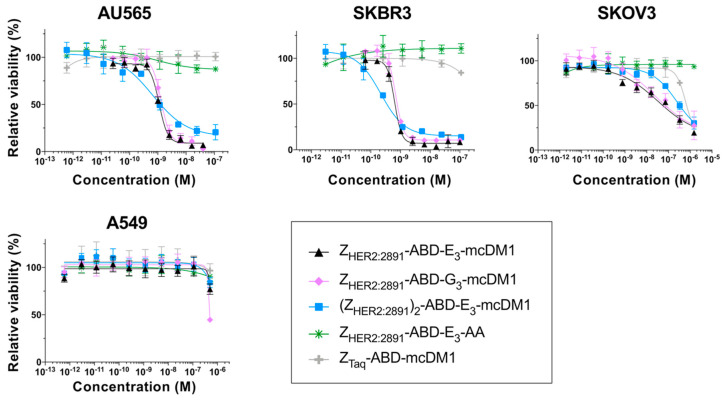
In vitro cytotoxicity analysis of the conjugates. The cytotoxicity was determined by incubating serial dilutions of the conjugates with the cell lines indicated above the panels. The relative viability of the cells is plotted on the Y-axis as a function of the conjugate concentration on the X-axis. The relative viability of cells cultivated in complete medium was used for normalization to 100% viability. Each datapoint corresponds to the average of four independent experiments and the error bars correspond to 1 SD.

The cytotoxicities of the AffiDCs were measured by treating AU565 (high HER2 expression), SKBR3 (high HER2 expression), SKOV3 (high HER2 expression), and A549 (moderate HER2 expression) cells with serial dilutions of the conjugates followed by measurement of cell viability (Figure 2). Two controls were included: The non-toxic control Z_HER2:2891_-ABD-E_3_-AA lacking mcDM1, and the non-target control Z_Taq_-ABD-mcDM1, where Z_HER2:2891_ had been replaced with Z_Taq_, an affibody molecule that does not bind to any protein on the cell surface.

For all HER2-overexpressing cells, the AffiDCs showed a dose-dependent cytotoxic effect. The IC_50_ values ranged from 0.65 to 1.31 nM on AU565, from 0.22 to 0.69 nM for SKBR3, and from 23.5 to 245 nM on SKOV3 cells (Table 3). All conjugates demonstrated a substantially weaker cytotoxic effect on A549 cells. For this cell line, the IC_50_ values could not be measured at the concentrations used, but from Figure 2, it is evident that they were weaker than 10^−6^ M in all cases. The non-toxic control Z_HER2:2891_-ABD-E_3_-AA did not affect viability of any of the cell lines at any of the concentrations tested. For all four cell lines, the non-target control Z_Taq_-ABD-mcDM1 required high concentrations before a reduction in viability could be detected. The IC_50_ values could not be determined from the concentration range used, except for SKOV3 cells where the IC_50_ value was 619 nM. No significant difference in IC_50_ values was found when comparing Z_HER2:2891_-ABD-E_3_-mcDM1 and Z_HER2:2891_-ABD-G_3_-mcDM1, differing only by the linker (E_3_ or G_3_). A comparison of the two monovalent AffiDCs, with the dimeric construct, (Z_HER2:2891_)_2_-ABD-E_3_-mcDM1, showed that all three had similar IC_50_ values. However, the curves for the divalent construct were shallower than the curves for the monovalent constructs.

### 2.4. Labeling with ^99m^Tc

In preparation for determination of cell interaction and biodistribution, the AffiDCs, Z_HER2:2891_-ABD-E_3_-mcDM1, Z_HER2:2891_-ABD-G_3_-mcDM1, and (Z_HER2:2891_)_2_-ABD-E_3_-mcDM1, were labeled with ^99m^Tc. The labeling was site-specifically directed to a tag with the amino acid sequence His-Glu-His-Glu-His-Glu in the N-terminus of the conjugates. This labeling approach provides a residualizing label, which allows for tracking of a conjugate’s uptake and catabolism while having a minimal impact on its binding properties. The characteristics of the radiolabeled AffiDCs are presented in Table S1. The radiochemical yield in the labeling reaction ranged from 94% to 98%, and after purification, the radiochemical purity was >99.5% for all three conjugates. No significant release of activity was observed in a stability test, where the radiolabeled conjugates were challenged with a high concentration of histidine.

### 2.5. Cell Binding and Cellular Processing

**Figure 3:**
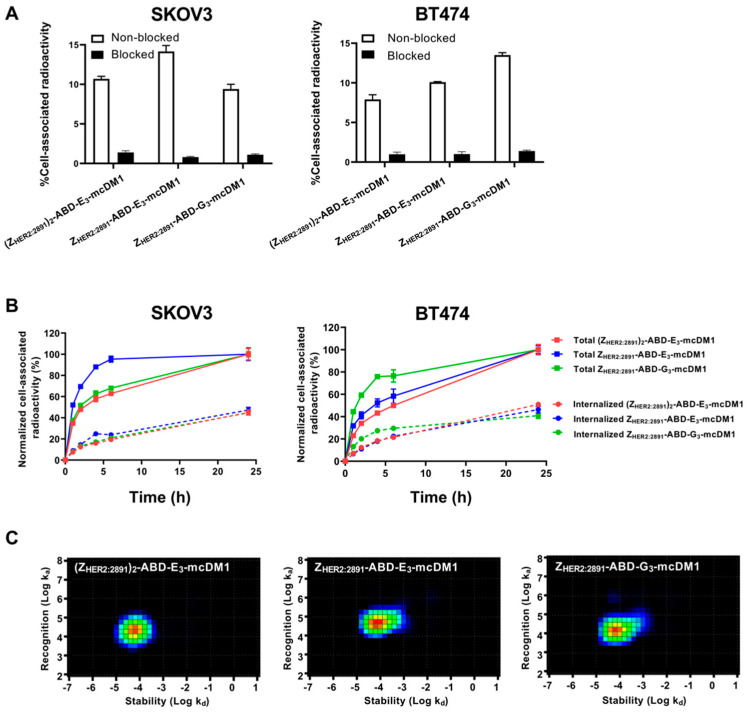
Cell binding and cellular processing of ^99m^Tc-labeled AffiDCs. (**A**) SKOV3 and BT474 cells were incubated with radiolabeled AffiDC with or without pre-saturation with a 500-fold molar excess of the same AffiDC lacking radiolabel, blocked or non-blocked, respectively. The Y-axis corresponds to the measured total radioactivity of the cells as a percentage of the total radioactivity added to each well. (**B**) Cellular processing of 2 nM conjugates by SKOV3 and BT474 cells during continuous incubation over 24 h. Data were normalized to the recorded value at 24 h, which was set to 100%, and are presented as mean with error bars corresponding to 1 SD. (**C**) Interaction maps for the interaction between ^99m^Tc labeled AffiDCs and SKOV3 cells. The maps were derived by InteractionMap software from real-time biosensor measurements in a LigandTracer instrument. The x-axis corresponds to the dissociation rates and the y-axis corresponds to the association rates of the interactions in each graph.

To investigate cell binding specificity, the high HER2-expressing cell lines, SKOV3 and BT474, were incubated with ^99m^Tc labeled conjugates, with or without previous blocking of available HER2 receptors by a 500-fold molar excess of the same non-radiolabeled conjugate. The result showed a significantly decreased cell-associated radioactivity (*p* < 0.05) in the blocked groups for all conjugates in both cell lines (Figure 3A). The results indicated specific HER2-mediated binding of all ^99m^Tc-labeled conjugates to SKOV3 and BT474 cells.

In order to investigate cellular uptake and processing of the conjugates, SKOV3 and BT474 cells were incubated with radiolabeled conjugates and the level of membrane-bound and internalized radioactivity was measured as a function of time (Figure 3B). The total cell-associated radioactivity increased for all conjugates on both cell lines for the duration of the experiment (24 h), except for Z_HER2:2891_-ABD-E_3_-mcDM1, which associated more rapidly (95% after 6 h incubation) with SKOV3 cells. Z_HER2:2891_-ABD-G_3_-mcDM1 associated more rapidly (80% after 6 h incubation) with BT474 than the other two. After incubation for 24 h, the internalized fraction accounted for 30% to 40% of the total cell-associated radioactivity for the three conjugates in both cell lines.

The binding kinetics of the radiolabeled conjugates to SKOV3 cells was also measured in real-time on a LigandTracer instrument followed by analysis by InteractionMap software. The equilibrium dissociation constants (K_D_) values were 1.76 ± 0.03, 5.4 ± 0.2, and 3.6 ± 0.6 nM for Z_HER2:2891_-ABD-E_3_-mcDM1, Z_HER2:2891_-ABD-G_3_-mcDM1, and (Z_HER2:2891_)_2_-ABD-E_3_-mcDM1, respectively. The analyzes showed single interactions in all three cases, even though (Z_HER2:2891_)_2_-ABD-E_3_-mcDM1 contains two HER2-binding domains (Figure 3C).

### 2.6. Biodistribution

**Figure 4:**
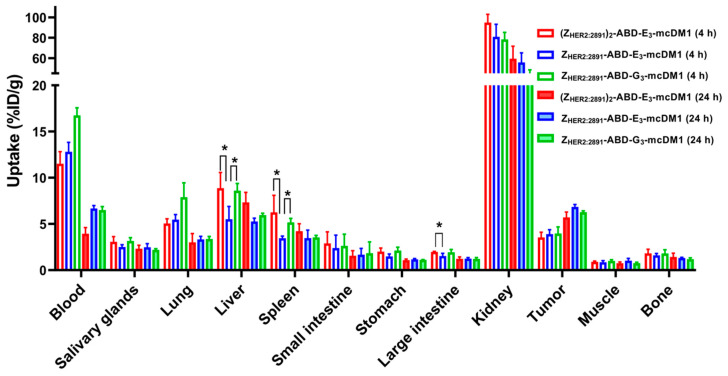
Biodistribution of ^99m^Tc-labeled AffiDCs in female BALB/c nu/nu mice with SKOV3 xenografts at 4 and 24 h after injection. The radioactivity is expressed as per cent of injected dose per gram tissue (%ID/g), and presented as an average value from 3 to 4 animals ± 1 SD. Star signs (*) correspond to significant differences (*p* < 0.05).

The biodistribution of ^99m^Tc-labeled conjugates was studied in mice bearing SKOV3 xenografts at 4 and 24 h p.i. (Figure 4, Table S2). At both time points, the highest uptake of all conjugates was in kidneys. The radioactivity accumulated in blood was over 11% at 4 h p.i. and over 4% at 24 h p.i. This indicated a noticeable effect of including the ABD for prolonged blood retention. At 4 h p.i. the radioactivity uptake in the tumors was similar for all three conjugates. The monovalent Z_HER2:2891_-ABD-E_3_-mcDM1 had significantly (*p* < 0.05) lower uptake in liver and spleen compared to the other two, (Z_HER2:2891_)_2_-ABD-E_3_-mcDM1 and Z_HER2:2891_-ABD-G_3_-mcDM1. It also had a significantly (*p* < 0.05) lower uptake in large intestine compared to (Z_HER2:2891_)_2_-ABD-E_3_-mcDM1. However, at 24 h p.i. there was no significant difference in uptake between the three conjugates in tumor or in normal organs. At 4 h after injection, the tumor uptakes of all three tested conjugates were significantly (*p* < 0.05) lower than their uptakes in lung and liver. At 24 h, the tumor uptakes of (Z_HER2:2891_)_2_-ABD-E_3_-mcDM1 and Z_HER2:2891_-ABD-G_3_-mcDM1 were significantly (*p* < 0.05) higher than their uptake in lung. The differences between uptakes of these conjugates in tumor and liver were not significant. The tumor uptake of Z_HER2:2891_-ABD-E_3_-mcDM1 was significantly (*p* < 0.05) higher than its uptake in liver and lung at 24 h p.i.

**Figure 5:**
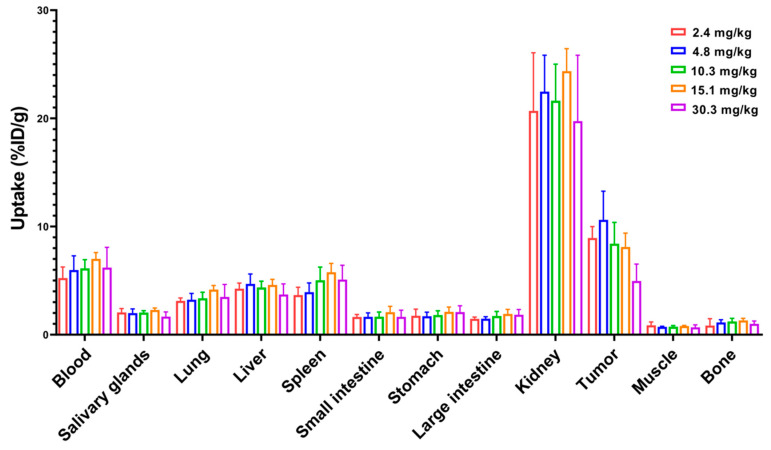
Dose escalation experiment where ^99m^Tc-labeled Z_HER2:2891_-ABD-E_3_-mcDM1 was injected into female BALB/c nu/nu mice carrying SKOV3 xenografts. The radioactivity in each organ was measured 48 h p.i. and is expressed as per cent of injected dose per gram tissue (%ID/g), and presented as an average value from 3 to 5 animals ± 1 SD.

Next, a dose escalation experiment was performed to investigate the influence of injected dose on biodistribution. The experiment was carried out by injecting increasing doses (2.4 to 30.3 mg/kg) of ^99m^Tc-labeled Z_HER2:2891_-ABD-E_3_-mcDM1 in mice bearing SKOV3 xenografts and measuring uptake at 48 h p.i. (Figure 5). There was no significant difference in uptake in normal organs at different doses. Since the measured uptake is based on percent of the injected dose per gram, the absolute values for uptake in different organs increased linearly with increasing dose. For uptake in tumor, there was no significant difference in uptake for doses up to 15.1 mg/kg. However, increasing the dose to 30.3 mg/kg resulted in significant (*p* < 0.05) decrease in tumor uptake. This shows that there was a linear increase in the absolute uptake in the tumor up to an injected dose of 15.1 mg/kg, after which the tumors began to be saturated.

### 2.7. Therapy Study

**Figure 6:**
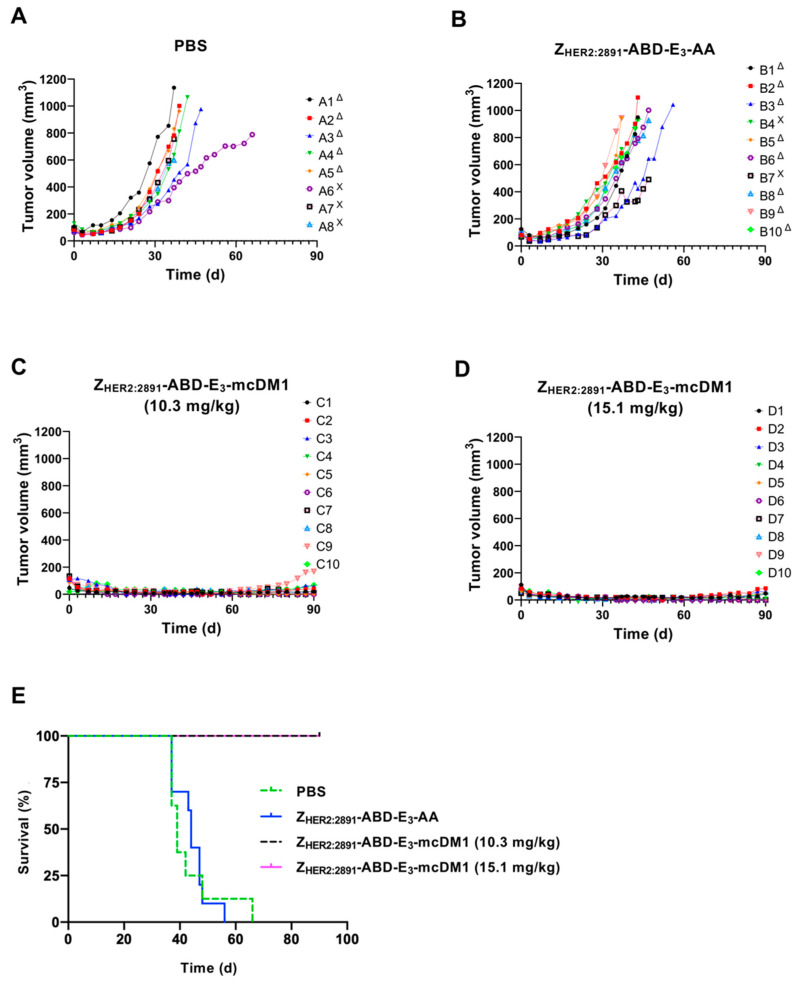
Experimental therapy with tumor volume growth curves for individual mice in each group. SKOV3 cells were subcutaneously implanted into the belly of nude BALB/c nu/nu mice. One week later the mice were randomized into four groups and given s.c. injections (scruff of the neck) of either PBS (**A**), 15.1 mg/kg Z_HER2:2891_-ABD-E_3_-AA (**B**), 10.3 mg/kg Z_HER2:2891_-ABD-E_3_-mcDM1 (**C**), or 15.1 mg/kg Z_HER2:2891_-ABD-E_3_-mcDM1 (**D**). Four injections were given at day 0, 7, 14, and 21. The mice were euthanized when the volume of the subcutaneous xenografts exceeded 1000 mm^3^ (Δ) or bleeding ulcers on the xenografts were observed (Χ). (**E**) Survival of the mice during the experimental therapy.

To investigate the therapeutic effect of Z_HER2:2891_-ABD-E_3_-mcDM1 in a mouse model of HER2-expressing tumors, mice bearing SKOV3 tumors were treated with four weekly injections on days 0, 7, 14, and 21, at two different concentrations (10.3 and 15.1 mg/kg) of the conjugate. Two control groups were included where one received injections of only buffer (PBS) and one received the non-toxic control Z_HER2:2891_-ABD-E_3_-AA (15.1 mg/kg). At 10 days after the first injection, a significant difference in tumor volume between both treatment groups and the control groups was found (Figure 6A). All animals in both treatment groups survived to the end of the experiment (90 days). In contrast, the median survival of the animals in the control groups were 39 d (PBS) and 44 d (Z_HER2:2891_-ABD-E_3_-AA). By day 66, all animals in the control groups had reached the humane endpoint and had thus been removed. The weight of the animals was measured and was not significantly affected by injection of 10.3 mg/kg Z_HER2:2891_-ABD-E_3_-mcDM1 compared to both control groups. For the highest dose treatment group (15.1 mg/kg Z_HER2:2891_-ABD-E_3_-mcDM1) a tendency for weight loss was detected. In this group, the average weight decreased after the third injection from 16.8 ± 1.7 g to 15.5 ± 2.4 g but increased to a non-significant difference, 10 days after the fourth injection. At the end of the study, two mice in the 10.3 mg/kg and three mice in 15.1 mg/kg groups did not have any observable tumors.

**Figure 7:**
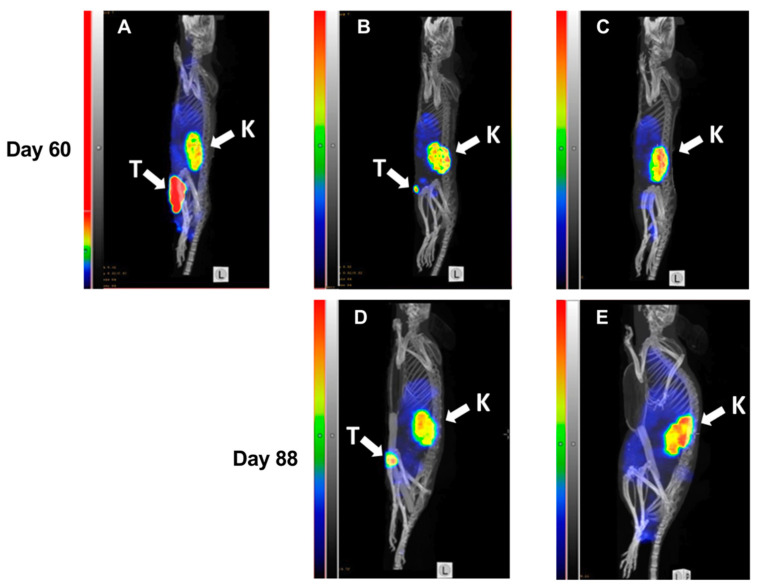
MicroSPECT/CT imaging of HER2-expression in BALB/c nu/nu mice bearing SKOV3 xenografts. Maximum Intensity Projections, sagittal view. Imaging was performed at days 60 (**A**–**C**) and 88 (**D**,**E**) of the experimental therapy. A mouse from the PBS-treated control group with a large necrotic tumor (**A**). A mouse with a small tumor from the group treated with Z_HER2:2891_-ABD-E_3_-mcDM1 (15.1 mg/kg) at day 60 (**B**) and at day 88 (**D**). A mouse without any visible tumor from the group treated with Z_HER2:2891_-ABD-E_3_-mcDM1 (10.3 mg/kg) at day 60 (**C**) and at day 88 (**E**). The relative scale in (**A**) was adjusted to the same level of activity in kidneys for (**B**–**E**).

At day 60, SPECT/CT imaging of HER2 expression using ^99m^Tc-labeled Z_HER2:V2_ was performed (Figure 7) and the resulting images were in accordance with tumor volume measurement results. Both a large (Figure 7A) as well as a small (Figure 7B) tumor could be visualized by the radiotracer. The results of imaging at day 60 were predictive for the treatment outcome. The mouse with the large tumor at day 60 (Figure 7A) was euthanized five days after the image was taken because of tumor ulceration. There was some increase in size of the small tumor from day 60 to day 88 (Figure 7B). The mouse without tumor (Figure 7C) at day 60 remained tumor-free during next imaging session at day 88 and at the termination of the study.

After removal of the mice from the study, a histopathological examination of the liver and kidney was performed in each animal. No pathological changes were found in any of the animals, including the animals receiving the dose of 15.1 mg/kg.

## 3. Discussion

Patients with HER2-overexpressing metastatic breast cancer are normally prescribed treatment with HER2-targeting drugs. However, in most instances, this is not a curative treatment and patients eventually progress. Even though resistance may not be attributed to the mAb or ADC format, novel drugs are in need that may either be used in combination with standard drugs or even replace them. This motivated us to investigate the affibody-class of engineered affinity proteins (EAPs) as carriers of potent cytotoxic drugs, creating so called affibody drug conjugates (AffiDCs).

The AffiDCs generated in this study were relatively simple to produce, where recombinantly produced fusion proteins in *Escherichia coli* could be purified with a single affinity chromatography step, before mcDM1 conjugation, followed by a high-resolution RP-HPLC purification step after conjugation. The resulting conjugates were highly pure and showed no tendency to aggregate (Figure 1). A simple and efficient manufacturing process is desirable and is advantageous compared to the typical production processes of ADCs, which usually require more advanced host organisms and where the final high-resolution RP-HPLC purification step cannot be employed, since it would irreversibly denaturate the mAb-part of the ADC. In addition, many ADCs in clinical development employ random attachment of the drug to lysines or cysteines, in contrast to the AffiDCs in this study, where mcDM1 was site-specifically attached to the C-terminal cysteine in all cases, resulting in homogenous substances.

An interesting observation was the difference in Hill slopes between the di- and monovalent conjugates in the in vitro analysis of cytotoxic potential (Figure 2). The shallower slopes of the divalent construct on AU565 and SKBR3 cells could indicate that there is negative cooperativity in binding to the cells compared to the monovalent constructs [24]. However, for SKOV3 cells, the Hill slopes for the di- and the monovalent variants were similar. This shows that even though there could be negative or positive cooperativity in binding, the resulting cellular effect might be influenced by additional parameters. A possible reason could be that it is not only cell binding that is responsible for toxic action but that also other pathways are involved. This has been studied in some detail for ADC toxicity where differences in the rate of receptor internalization, expression level of multi-drug resistance proteins and impairment of lysosomal degradation are different between different cell lines [4,25,26]. Such parameters likely affect the sensitivity to Z_HER2:2891_-ABD-E_3_-mcDM1 and this leads to differences in IC_50_-values among the cell lines with high HER2 expression in this study. However, it is unclear why these parameters would affect the Hill slope. Differences in Hill slopes and sensitivity to ADCs have also previously been observed in dose-response curves for T-DM1 on different cell lines [27].

In vivo, the biodistribution experiment (Figure 4) showed that even small changes in the carrier protein affect the uptake in different organs. Since Z_HER2:2891_ does not cross-react with murine HER2, the uptake in normal organs is not mediated by HER2 and is only a consequence of non-specific uptake. At 4 h p.i., the uptake in liver of Z_HER2:2891_-ABD-E_3_-mcDM1 was significantly lower than the uptake of Z_HER2:2891_-ABD-G_3_-mcDM1. These conjugates were identical except for that the tri-glutamyl linker had been replaced with a tri-glycine linker. Interestingly, the uptake in liver of Z_HER2:2891_-ABD-E_3_-mcDM1 was also significantly lower than the divalent (Z_HER2:2891_)_2_-ABD-E_3_-mcDM1, suggesting that the Z_HER2:2891_ part promotes uptake. The plasma half-life was around 20 h for all three conjugates, which was lower than for example Z_HER2:342_-ABD conjugated with ^177^Lu [28]. Even though they cannot be directly compared since the mcDM1 and ^177^Lu parts may promote clearance differently, it would be interesting to investigate other methods for half-life extension of AffiDCs such as PEGylation [29] and PASylation [30] to learn if an even longer plasma half-life would lead to even more efficacious drug delivery to the tumor. In contrast to ADCs, the AffiDCs allow for detailed investigation of the impact of plasma half-life on drug candidate performance. ADCs, on the other hand, always have a relatively long plasma half-life, and for them a long half-life is generally regarded as beneficial [31]. A translation of Z_HER2:2891_-ABD-E_3_-mcDM1 from mouse to human would likely lead to a longer plasma half-life since the plasma half-life of HSA is longer than MSA and the affinity of the ABD is stronger for HSA than for MSA.

Previously, we found that a divalent format of an AffiDC, (Z_HER2:2891_)_2_-ABD-mcDM1, was able to slow tumor growth and as a consequence increase survival of mice with implanted HER2-overexpressing SKOV3 tumors [13]. However, when determining the cytotoxic potential on HER2-overexpressing cells in vitro in a dose-response experiment, there was no significant difference of the IC_50_ values of the di- and monovalent constructs. Similarly, in this study the difference in IC_50_ values between di- and monovalent variants was not significant. Since a smaller construct will theoretically penetrate solid tumors better than a larger one [6], one of the aims of this study was to evaluate the efficacy of the smaller monovalent conjugate. The experimental therapy data cannot be directly compared between the divalent (Z_HER2:2891_)_2_-ABD-mcDM1 and the monovalent Z_HER2:2891_-ABD-E_3_-mcDM1 in present study, since the monovalent construct included an E_3_ linker next to the cysteine onto which mcDM1 was attached. The injected dose was also higher in the present study (15.1 and 10.3 mg/kg), which corresponds to 1.11 μmol/kg or 0.76 μmol/kg compared to the previous study where the dose was 0.41 μmol/kg. However, a strikingly more efficient therapy was detected in the present study, with efficient regression of all tumors and complete disappearance of the xenografts in some animals treated with Z_HER2:2891_-ABD-E_3_-mcDM1. At the highest dose (15.1 mg/kg), we found that 2 out of 10 animals started to lose weight after three weekly injections, which suggested that this was close to the maximum tolerated dose. One interesting aspect of this and our previous study [13] is that the histology investigation did not reveal pathologic changes in the kidneys although the renal uptake was higher than the tumor uptake. There are similar observations with other engineered scaffold proteins, conjugated with microtubule polymerization inhibitory drugs, e.g., conjugates of PASylated DARPin with MMAF [30,32] and Bicycles conjugate with MMAE [12]. In these cases, a strong anti-tumor effect was observed without a pronounced effect on the kidneys, even though the renal uptake was higher that the tumor uptake. It has to be noted that these drugs are inhibiting microtubule polymerization and are likely more toxic to rapidly dividing cells in the tumor than to slower dividing cells in normal tissues.

Z_HER2:2891_-ABD-E_3_-mcDM1 could be interesting to use in combination with already established drugs, such as T-DM1. One reason is that the pharmacokinetic profile is different with faster clearance of Z_HER2:2891_-ABD-E_3_-mcDM1 compared to T-DM1. A second reason is that the uptake in normal organs is different. For example, ^89^Zr-labeled T-DM1 has a higher uptake in spleen and bone [33] compared to ^99m^Tc-labeled Z_HER2:2891_-ABD-E_3_-mcDM1, whereas ^99m^Tc-labeled Z_HER2:2891_-ABD-E_3_-mcDM1 has a higher uptake in kidneys. A combination of T-DM1 and Z_HER2:2891_-ABD-E_3_-mcDM1 would therefore likely allow for delivery of a higher total dose of DM1 to the tumor, while the unspecific uptake in spleen, bone, and kidney could be kept on a manageable level. However, side effects associated with T-DM1 such as severe thrombocytopenia (grade ≥ 3) [34] requires careful studies to elucidate if a combination of Z_HER2:2891_-ABD-E_3_-mcDM1 and T-DM1 is superior to either of the conjugates alone. It should also be noted that the antibody part of an ADC delivers some of its therapeutic effect by acting as a HER2 antagonist and by antibody-dependent cellular cytotoxicity (ADCC) [35]. These therapeutic mechanisms are not available to the monomeric affibody carrier in this article.

ADCs are often predominantly cleared through the liver and the second most common grade 3 adverse effect in patients receiving T-DM1 is elevation of liver enzymes including aspartate and alanine aminotransferases [36]. Our results show that clearance through the kidneys is the predominant route for Z_HER2:2891_-ABD-E_3_-mcDM1, which could be beneficial if the kidneys are more resistant to the cytotoxic effect of DM1. Radiolabeled compounds could prove to be beneficial in a combination treatment strategy including Z_HER2:2891_-ABD-E_3_-mcDM1 and e.g., the analogous Z_HER2:342_-ABD conjugate labeled with ^177^Lu [28] or a pre-targeting concept involving ^177^Lu labeled PNA [37]. Since Z_HER2:342_ interacts with the same epitope as Z_HER2:2891_, the injected dose of Z_HER2:2891_-ABD-E_3_-mcDM1 has to be lower than the saturating concentration (15.3 mg/kg in the experiment presented in Figure 5).

The composition of the linker is an important part of drug conjugates and for ADCs, the linkage is often a weak link in the design. The maleimide-thiol bond employed in T-DM1 as well as the AffiDCs in the present study has an in vivo half-life of only 24 h, and the lability of this bond may cause more release of free mcDM1 and cross-transfer to endogenous proteins [38] for the ADC than for the AffiDC due to its longer plasma half-life. However, strategies to increase linker stability have been described, such as hydrolytic opening of the thiosuccinimide ring [38].

Z_HER2:2891_-ABD-E_3_-mcDM1 is a promising prototype where every affibody molecule is conjugated with a single drug moiety. One of the interesting directions for further improvement of AffiDC would be development of constructs capable of delivering several drugs. One of the challenges with this approach is that coupling of multiple hydrophobic drugs to a relatively small protein might have an adverse effect on its biodistribution. It has been demonstrated that an increase of lipophilicity of affibody molecules is associated with significantly elevated hepatic uptake [39], particularly when a lipophilic moiety is placed at the N-terminus. The finding in this study that a drug conjugate consisting of a monomeric affibody carrier in combination with a lipophilic drug, connected by a negatively charged and hydrophilic linker, has reduced hepatic uptake, is an essential input information for molecular design of the next-generation of AffiDCs. The next-generation AffiDCs might include more potent payloads at a higher multiplicity and would be interesting to compare head-to-head with T-DM1 and other clinically approved HER2 targeting drugs.

A big potential of radionuclide molecular imaging for enhancing efficacy of HER2-targeting therapies is well-recognized [40]. In the case of targeted delivery of cytotoxic drugs, the most apparent application is the stratification of patients according to HER2 expression level in tumors, to identify patients that would benefit from targeted therapy. Clinical studies suggest that trastuzumab emtansine is the most efficient in the case of tumors with high HER2 expression [41,42] and it is thus important to identify those patients. It has been demonstrated that a combination of SPECT/CT imaging using [^18F^]2-fluoro2-deoxy-D-glucose (FDG) and ^89^Zr-labeled trastuzumab can predict the radiologic response and the median time to treatment failure for T-DM1 therapy [43]. Our study indicated that radionuclide imaging may be used for monitoring of response to Z_HER2:2891_-ABD-E_3_-mcDM1-mediated therapy (Figure 7), and that it can also be utilized for stratification purposes. The use of an affibody molecule as a theranostic companion to Z_HER2:2891_-ABD-E_3_-mcDM1 is preferable to the use of radiolabeled antibodies, as it also indicates an accessibility of the targeted epitope.

## 4. Materials and Methods

### 4.1. General

All chemicals were purchased from Sigma-Aldrich (St. Louis, MO, USA) or Merck (Darmstadt, Germany) unless otherwise stated. Restriction enzymes were from New England Biolabs (Ipswitch, MA, USA).

### 4.2. Construction of Genes

Genes encoding Z_HER2:2891_-ABD-E_3_-Cys and Z_HER2:2891_-ABD-G_3_-Cys, flanked by NdeI and BamHI restriction sites, were amplified from a plasmid encoding (Z_HER2:2891_)_2_-ABD-Cys [13] using Phusion polymerase (New England Biolabs). Likewise, the gene encoding Z_Taq_-ABD-Cys was amplified from a plasmid encoding (Z_Taq_)_2_-ABD-Cys [13]. During PCR amplification, a tag with the amino acid sequence His-Glu-His-Glu-His-Glu (HEHEHE) was placed at the N-terminus of all constructs allowing for radionuclide labeling. The linker connecting the affibody molecules and ABD had the amino acid sequence Gly-Gly-Gly-Gly-Ser. The genes were sub-cloned into the pET-21a(+) plasmid vector (Novagen, Madison, WI, USA) using NdeI and BamHI restriction enzymes. The expression vector encoding (Z_HER2:2891_)_2_-ABD-E_3_-Cys was constructed previously [21]. The sequences of all genes were confirmed by DNA sequencing.

### 4.3. Expression and Purification

The affibody constructs were expressed at 37 °C in shake flask cultures of *Escherichia coli* BL21 Star (DE3) (New England Biolabs) in tryptic soy broth containing 100 µg/mL ampicillin. When OD_600_ was between 0.6 and 1, protein expression was induced by addition of 1 mM isopropyl-β-D-1-thiogalactopyranoside (Appolo Scientific, Stockport, UK). Protein production was carried out for 3 h, after which the cells were harvested by centrifugation and lysed by sonication. The supernatants were clarified by centrifugation and filtration through a 0.45 µm Acrodisc syringe filter (Pall, Port Washington, NY, USA). The recombinantly expressed affibody constructs were purified by affinity chromatography on a HiTrap NHS sepharose column (GE Healthcare, Uppsala, Sweden) with immobilized human serum albumin (HSA) using an ÄKTA system (GE Healthcare), essentially as previously described [44] including elution with 500 mM acetic acid, pH 2.6. The fractions containing affibody constructs were pooled and lyophilized.

Z_HER2:V2_ was produced and purified as previously described [45].

### 4.4. Conjugation with mcDM1

The lyophilized proteins were dissolved in 100 mM Tris-HCl buffer (pH 7.85) to a final concentration of 0.1 mM. Subsequently, the proteins were incubated with 5 mM tris (2-carboxyethyl) phosphine (TCEP) for 30 min at 37 °C to reduce the thiol group on the C-terminal cysteine of the constructs, which could potentially have been oxidized during protein production and purification. Before mcDM1 was added, the pH of protein solution was adjusted to 6.5 using 1 M HCl. A freshly prepared solution of mcDM1 (Levena Biopharma, San Diego, CA, USA), in DMSO (20 mM), was mixed with the affibody constructs at a molar ratio of 2:1 (drug: protein), and the conjugation mixture was incubated overnight at room temperature. The conjugation reaction mixture was diluted with HPLC buffer A (0.1% trifluoroacetic acid in H_2_O) and then loaded on a Zorbax SB-C18 column (Agilent, Santa Clara, CA, USA). Bound material was eluted by a 30 min gradient from 30% to 60% buffer B (0.1% trifluoroacetic acid in acetonitrile) at a flowrate of 3 mL/min. The fractions containing affibody-mcDM1 conjugates were pooled followed by lyophilization. Capping of the C-terminal cysteine to create the non-toxic control Z_HER2:2891_-ABD-E_3_-AA was carried out with 2-iodoacetamide. Lyophilized Z_HER2:2891_-ABD-E_3_-Cys was dissolved in alkylation buffer (0.2 M NH_4_HCO_3_, pH 8.0) potentially oxidized cysteine residues were reduced with TCEP as above. 2-iodoacetamide was added to a final concentration of 10 mM followed by incubation for 30 min at room temperature in the dark to alkylate cysteines. The capped proteins were purified by RP-HPLC as described above for the AffiDCs, followed by lyophilization. The AffiDCs and the non-toxic control were dissolved in phosphate buffered saline (PBS, 10 mM Na-phosphate, 2.7 mM KCl, 137 mM NaCl, pH 7.4) and stored at −80 °C until use.

Purified conjugates (5 µg in each sample) were analyzed by SDS-PAGE (Biorad, Hercules, CA, USA) under reducing conditions. The oligomeric state of the conjugates was determined by size exclusion chromatography, by passage through a 5/150 column (GE Healthcare), packed with Superdex-75, at a flowrate of 0.45 mL/min in PBS. The molecular weight of purified affibody-mcDM1 conjugates was measured by ESI-TOF mass spectrometry (Agilent). Analysis of purity was measured by RP-HPLC using a Zorbax 300SB-C18 column (Agilent) using a gradient from 30% to 60% of HPLC buffer B during 20 min at a flow rate of 1 mL/min.

### 4.5. Binding Specificity and Affinity Determination

A Biacore 3000 instrument (GE Healthcare) was used for biosensor analysis. HER2-Fc chimeric protein (R&D Systems, Minneapolis, MN, USA) was immobilized to 696 RUs on a CM5 chip by amine coupling in sodium acetate buffer, pH 4.5. On a second CM5 chip, HSA (Novozymes, Bagsvaerd, Denmark) and MSA (Sigma-Aldrich) were immobilized in the same way. The final immobilization levels were 395 and 457 RUs, respectively. A reference flow cell was created by activation and deactivation on both chips. HBS-EP (10 mM HEPES, 150 mM NaCl, 3 mM EDTA, 0.005% v/v surfactant P20, pH 7.4) was used as running buffer and for dilution of the analytes. All experiments were performed at 25 °C at a flow rate of 50 µL/min. The chips were regenerated by injection of 10 mM HCl for 30 s. The binding kinetics were analyzed by Biacore evaluation software using the one-to-one kinetics model.

### 4.6. Cell Culture

AU565, SKBR3, SKOV3, BT474, and A549 cell lines were obtained from American Type Culture Collection (ATCC, via LGC Promochem, Borås, Sweden) and were grown in McCoy’s 5A (SKOV3, SKBR3), RPMI-1640 (AU565, BT474) or Dulbecco’s modified Eagle medium (A549) (Cytiva Hyclone, Uppsala, Sweden) supplemented with 10% FBS (20% FBS for BT474) (Sigma-Aldrich) in a humidified incubator at 37 °C in 5% CO_2_ atmosphere.

### 4.7. In Vitro Cytotoxicity Analysis

Approximately 5000 cells/well (2000 cells/well for SKOV3) were seeded in a 96-well plate and allowed to attach overnight. Subsequently, the medium was replaced with fresh medium containing serial dilutions of AffiDCs or controls, followed by incubation for 72 h. Cell viability was determined using Cell Counting Kit-8 (CCK-8, Sigma-Aldrich) according to the manufacturer’s protocol with measurement of A_450_ in each well. The obtained absorbance values were analyzed by GraphPad Prism using a log(inhibitor) vs. response-variable slope (four parameters) model (GraphPad Software, La Jolla, CA, USA).

### 4.8. Radiolabeling

Radiolabeling of the AffiDCs with ^99m^Tc, using [^99m^Tc(CO)_3_(H_2_O)_3_]^+^ precursor, was performed using a CRS kit (PSI, Villigen, Switzerland) according to manufacturer’s instructions. In brief, technetium-99m was freshly eluted as pertechnetate, ^99m^TcO_4_^−^, with 0.9% NaCl from a commercial ^99^Mo/^99m^Tc generator. To generate the [^99m^Tc(CO)_3_(H_2_O)_3_]^+^ (tricarbonyl technetium) precursor, 500 μL ^99m^TcO_4_^−^ was added to a CRS kit vial, followed by incubation at 100 °C for 30 min. A solution containing 60 μg of an AffiDC in 100 μL PBS was mixed with 60 MBq of [^99m^Tc(CO)_3_(H_2_O)_3_]^+^ and incubated at 60 °C for 60 min.

The radiolabeled AffiDCs were separated from [^99m^Tc(CO)_3_(H_2_O)_3_]^+^ by passage through a NAP-5 size exclusion chromatography column (GE Healthcare) eluted with 2% BSA in PBS. To evaluate the stability of labeling, the radiolabeled conjugates were challenged with 5000-fold molar excess of histidine in PBS for 4 h at room temperature. The radiochemical yield and purity were measured using iTLC analysis where the iTLC strips were eluted with PBS. Analysis of the iTLC strips were done using a Cyclone Storage Phosphor System (PerkinElmer, Waltham, MA, USA). The activity was measured using an automatic gamma spectrometer equipped with a 3-inch NaI(Tl) well detector (1480 Wizard, Wallac, Finland).

Labeling of Z_HER2:V2_ with ^99m^Tc was performed as previously described [45].

### 4.9. In Vitro Characterization of ^99m^Tc-Labeled AffiDCs

An in vitro specificity test was performed as described earlier [46]. In brief, SKOV3 and BT474 cells were seeded in 6-well plates at a density of 4 to 6 × 10^5^ cells/well, 1 or 2 days before the experiment. Non-radiolabeled AffiDC (1000 nM) was then added to each well or an equal volume of medium only to control wells followed by incubation for 15 min at room temperature. Then a solution of a radiolabeled AffiDC was added to each well to reach a final concentration of 2 nM followed by incubation for 1 h at 37 °C. Then, the medium was collected, and the cells were washed once with 1 mL PBS, detached from the well, and collected. The radioactivity of medium and cells was measured using a gamma spectrometer.

Evaluation of cellular processing was performed according to a method described earlier [46]. In brief, SKOV3 and BT474 cells were seeded (5 × 10^5^ cells/well) in 35 mm dishes 1 or 2 days before the experiment. The radiolabeled compound (2 nM) in medium was added and the dishes were incubated at 37 °C. At determined time points (1, 2, 4, 6 and 24 h after addition), the dishes were analyzed for membrane-bound and internalized radioactivity. Briefly, the medium from cells was collected followed by a wash with PBS. The cells were then treated with acid wash buffer (4 M urea in 0.2 M glycine, pH 2.0), for 5 min on ice to provide the membrane-bound fraction. Then the cells were incubated with 1 M NaOH at 37 °C for 30 min to release internalized radioactivity. The activity in every fraction was measured using an automatic gamma spectrometer.

The binding affinity of radiolabeled AffiDCs to HER2-expressing SKOV3 cells was evaluated using a LigandTracer Yellow instrument (Ridgeview Instruments, Vänge, Sweden) as described previously [47]. Approximately 2 × 10^6^ cells were seeded in 10 cm dishes 1 or 2 days before the experiment. To measure binding kinetics during the association phase, a stepwise addition of concentrations (1, 2 and 5 nM) of the AffiDCs in complete medium was performed. To measure dissociation, the medium was exchanged to complete medium lacking the AffiDC. Data were collected and analyzed using TraceDrawer software (Ridgeview Instruments), assuming a one-to-one kinetic model. The signal was corrected for nuclide decay. Analysis by InteractionMap software (Ridgeview Diagnostics, Uppsala, Sweden) was performed to estimate the interaction heterogeneity.

### 4.10. Biodistribution in Tumor Bearing Mice

SKOV3 xenografts were established in 22 female BALB/c nu/nu mice by implanting 12 × 10^6^ cells subcutaneously in a hind leg. The mice were randomized into 6 groups, 3 to 4 mice per group. Biodistribution of radiolabeled AffiDCs was measured 3 weeks after implantation. Mice were intravenously (i.v.) injected with 6 μg of ^99m^Tc-labeled conjugates in 2% BSA in PBS. The injected activity was calculated to be 60 kBq per mouse at the dissection time point. Before dissection, the mice were weighed and euthanized by an intraperitoneal injection of a ketamine-xylazine solution (30 μL of solution per gram body weight; ketamine 10 mg/mL; xylazine 1 mg/mL) and sacrificed by heart punctation. Blood, organs, and tissues were collected and weighed, and their radioactivity was measured.

An initial experiment to determine the dose of Z_HER2:2891_-ABD-E_3_-mcDM1 to be used for experimental therapy was performed. Briefly, SKOV3 xenografts were established in 28 female BALB/c nu/nu mice as described above. The mice were randomized and intraperitoneally injected with 2.4, 4.8, 10.3, 15.1, or 30.3 mg/kg of radiolabeled Z_HER2:2891_-ABD-E_3_-mcDM (5.12 MBq per animal) in 150–200 μL PBS. The biodistribution was measured after 48 h, as described above.

### 4.11. Therapy

An experiment to evaluate the therapeutic potential of Z_HER2:2891_-ABD-E_3_-mcDM1 was performed as follows: Female BALB/c nu/nu mice were subcutaneously implanted with 1×10^7^ SKOV3 cells in the abdomen. One week later, the mice were randomized to 4 groups (*n* = 8–10) and subcutaneously (scruff of the neck) injected with a test substance. This injection route was selected to avoid multiple injections in the same tail vein. Our previous studies have demonstrated that biodistribution of affibody molecules and their tumor uptake do not differ significantly already 4 h after subcutaneous or intravenous injections [48,49,50]. In treatment groups, either 10.3 or 15.1 mg/kg of Z_HER2:2891_-ABD-E_3_-mcDM1 in PBS were injected. Mice in control groups were injected with either PBS or with Z_HER2:2891_-ABD-E_3_-AA (15.1 mg/kg). The tumor volumes at the start of treatment were 86 ± 39, 80 ± 18, 96 ± 33, and 91 ± 22, mm^3^, for mice treated with PBS, Z_HER2:2891_-ABD-E_3_-AA, Z_HER2:2891_-ABD-E_3_-mcDM1 (10.3 mg/kg), and Z_HER2:2891_-ABD-E_3_-mcDM1 (15.1 mg/kg), respectively. Injection was done once a week for 4 consecutive weeks. Throughout the experiment, tumor volumes and body weights were monitored twice per week. The tumor volumes were determined by caliper measurement of the largest longitudinal (length) and transverse (width) diameter and calculated by the formula: Tumor volume = 1/2(length × width^2^).

Mice were euthanized when the subcutaneous tumor volume exceeded 1000 mm^3^, bleeding ulcers on the tumor were observed or 15% overall weight loss or 10% weight loss within one week was measured. The study was terminated 90 days after tumor implantation. After the mice were sacrificed, livers and kidneys were collected, fixated, and tested for histopathological changes. The histological examination was performed at the Department of Pathology and Wildlife Diseases, National Veterinary Institute, Uppsala, Sweden.

### 4.12. Imaging during Experimental Therapy

Three mice were selected for imaging using ^99m^Tc-labeled Z_HER2:V2_. Whole body micro-single photon emission computed tomography (SPECT)/CT scans were performed using nanoScan SPECT/CT (Mediso Medical Imaging Systems, Budapest, Hungary) at days 60 and 88. Mice were injected with ^99m^Tc labeled Z_HER2:V2_ (5 µg, 15–19 MBq) and a whole-body CT scan followed by a 10 min SPECT scan were performed 4 h after injection.

### 4.13. Statistics

Prism (version 8.4.3) was used for statistical analysis (GraphPad Software). Comparison of variation between multiple groups were carried out by one-way ANOVA with Bonferroni’s post hoc multiple comparisons test. Differences were considered significant when *p* < 0.05.

## 5. Conclusions

We have shown that drug conjugates utilizing a monovalent affibody molecule for targeting of the HER2 receptor leads to efficient regression of HER2 overexpressing human tumors implanted in mice. It is evident that changes in the protein part of the drug conjugate led to differences in the pharmacokinetic behavior, which necessitates careful design and investigation of multiple constructs to find the optimal protein part for combination with the particular drug at hand, which is also likely dependent on the cognate target of the affibody molecule.

## Figures and Tables

**Table 1:** Biochemical characterization of the AffiDCs.

Conjugate	Purity ^a^ (%)	Calc. MW (Da)	Found ^b^ MW (Da)
Z_HER2:2891_-ABD-E_3_-mcDM1	>95	14,383.8	14,383.3
Z_HER2:2891_-ABD-G_3_-mcDM1	>95	14,167.6	14,167.3
(Z_HER2:2891_)_2_-ABD-E_3_-mcDM1	>95	21,393.6	21,393.0
Z_HER2:2891_-ABD-E_3_-AA	>95	13,597.4	13,597.0

^a^ The purity was measured from the area under curve in the chromatograms in Figure 1D. ^b^ The molecular weights were determined by ESI-TOF mass spectrometry (Figure S1).

**Table 2:** Kinetic parameters and equilibrium dissociation constants.

Analyte	Ligand	k_a_ (M^−1^·s^−1^)	k_d_ (s^−1^)	K_D_ (M)
Z_HER2:2891_-ABD-E_3_-mcDM1	HER2	2.88 × 10^5^	2.10 × 10^−4^	7.28 × 10^−10^
	HSA	1.34 × 10^6^	7.50 × 10^−5^	5.58 × 10^−11^
	MSA	2.35 × 10^6^	2.07 × 10^−3^	8.97 × 10^−10^
Z_HER2:2891_-ABD-G_3_-mcDM1	HER2	4.04 × 10^5^	2.26 × 10^−4^	5.59 × 10^−10^
	HSA	1.09 × 10^6^	5.95 × 10^−5^	5.46 × 10^−11^
	MSA	1.92 × 10^6^	1.31 × 10^−3^	6.81 × 10^−10^
(Z_HER2:2891_)_2_-ABD-E_3_-mcDM1	HER2	ND ^a^	ND	ND
	HSA	1.51 × 10^5^	2.29 × 10^−5^	1.51 × 10^−10^
	MSA	2.08 × 10^5^	2.03 × 10^−3^	9.78 × 10^−9^
Z_HER2:2891_-ABD-E_3_-AA	HER2	8.09 × 10^5^	2.38 × 10^−4^	2.94 × 10^−10^
	HSA	1.73 × 10^6^	6.69 × 10^−5^	3.87 × 10^−11^
	MSA	2.82 × 10^6^	2.07 × 10^−3^	7.34 × 10^−10^

^a^ Not determined.

**Table 3:** In vitro cytotoxicity of the conjugates.

Cell Line	IC_50_ (nM)				
	Z_HER2:2891_-ABD-E_3_-mcDM1	Z_HER2:2891_-ABD-G_3_-mcDM1	(Z_HER2:2891_)_2_-ABD-E_3_-mcDM1	Z_HER2:2891_-ABD-E_3_-AA	Z_Taq_-ABD-mcDM1
AU565	1.11	1.31	0.65	ND ^a^	ND
SKBR3	0.64	0.69	0.22	ND	ND
SKOV3	32.9	23.5	245	ND	619

^a^ Not determined.

## Data Availability

The data generated during the current study are available from the corresponding author upon reasonable request.

## Supplementary Materials

The following are available online at https://www.mdpi.com/2072-6694/13/1/85/s1, Figure S1: Determination of the molecular masses by ESI-TOF mass spectrometry. Figure S2: Characterization of Z_Taq_-ABD-mcDM1. Figure S3: Biosensor analysis; Table S1: Yield, purity, and stability after ^99m^Tc-labeling; Table S2: Biodistribution of ^99m^Tc–labeled AffiDCs in tumor bearing mice.
