# Supplementary material for: Drug Conjugates Based on a Monovalent Affibody Targeting Vector Can Efficiently Eradicate HER2 Positive Human Tumors in an Experimental Mouse Model
Source: Cancers (Basel). 2020 Dec 30;13(1):85. doi: 10.3390/cancers13010085 (PMC7794879; doi:10.3390/cancers13010085)
Supplement: Supplementary file 1 [file cancers-13-00085-s001.zip › cancers-1010967-supplementary file-for conversion/cancers-1010967-supplementary-for proof.docx]

Article

Supplementary figures and tables: Drug conjugates based on a monovalent affibody targeting vector can efficiently eradicate HER2 positive human tumors in an experimental mouse model

Tianqi Xu, Haozhong Ding, Anzhelika Vorobyeva, Maryam Oroujeni, Anna Orlova, Vladimir Tolmachev and Torbjörn Gräslund


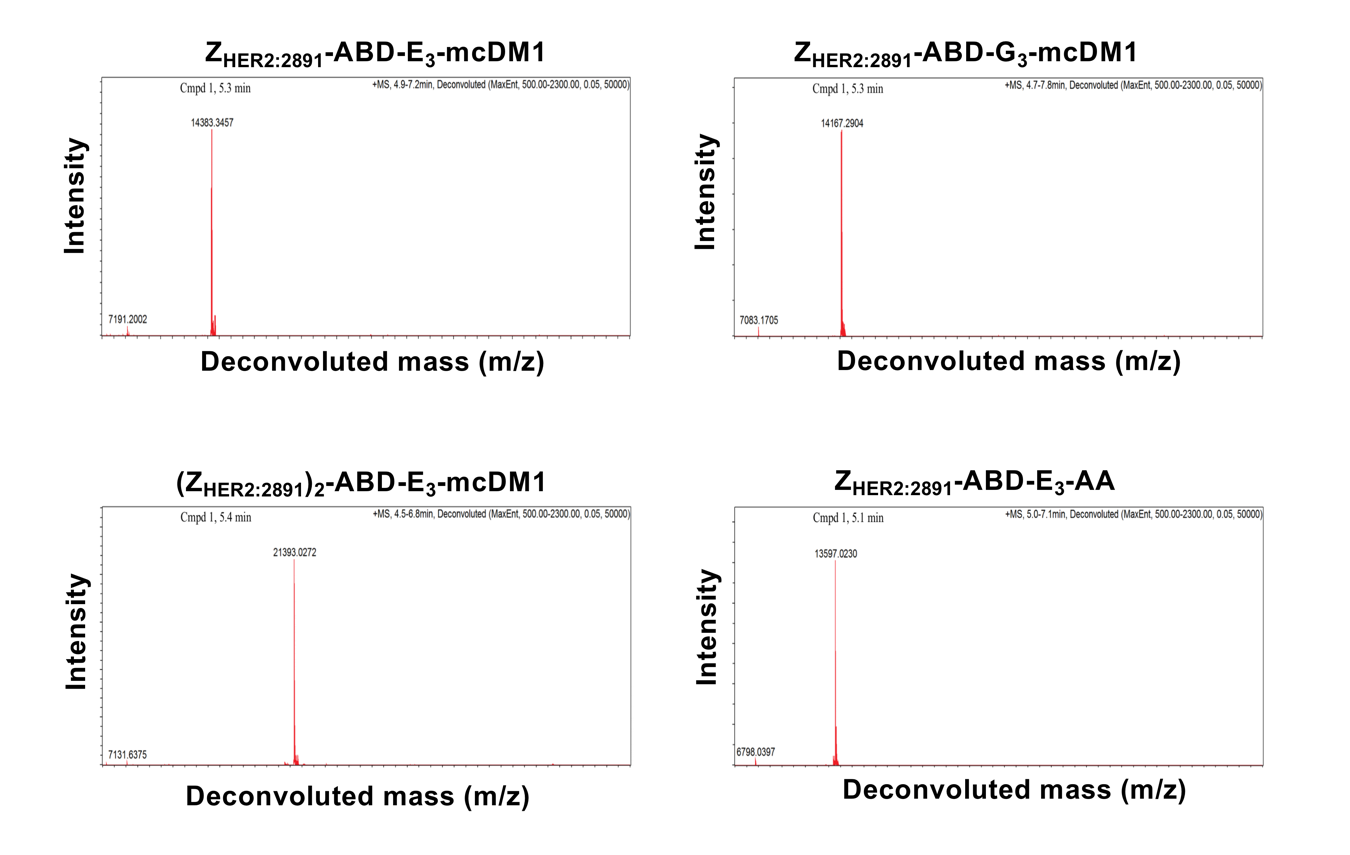


**Figure S1.** Determination of the molecular masses by ESI-TOF mass spectrometry.

**
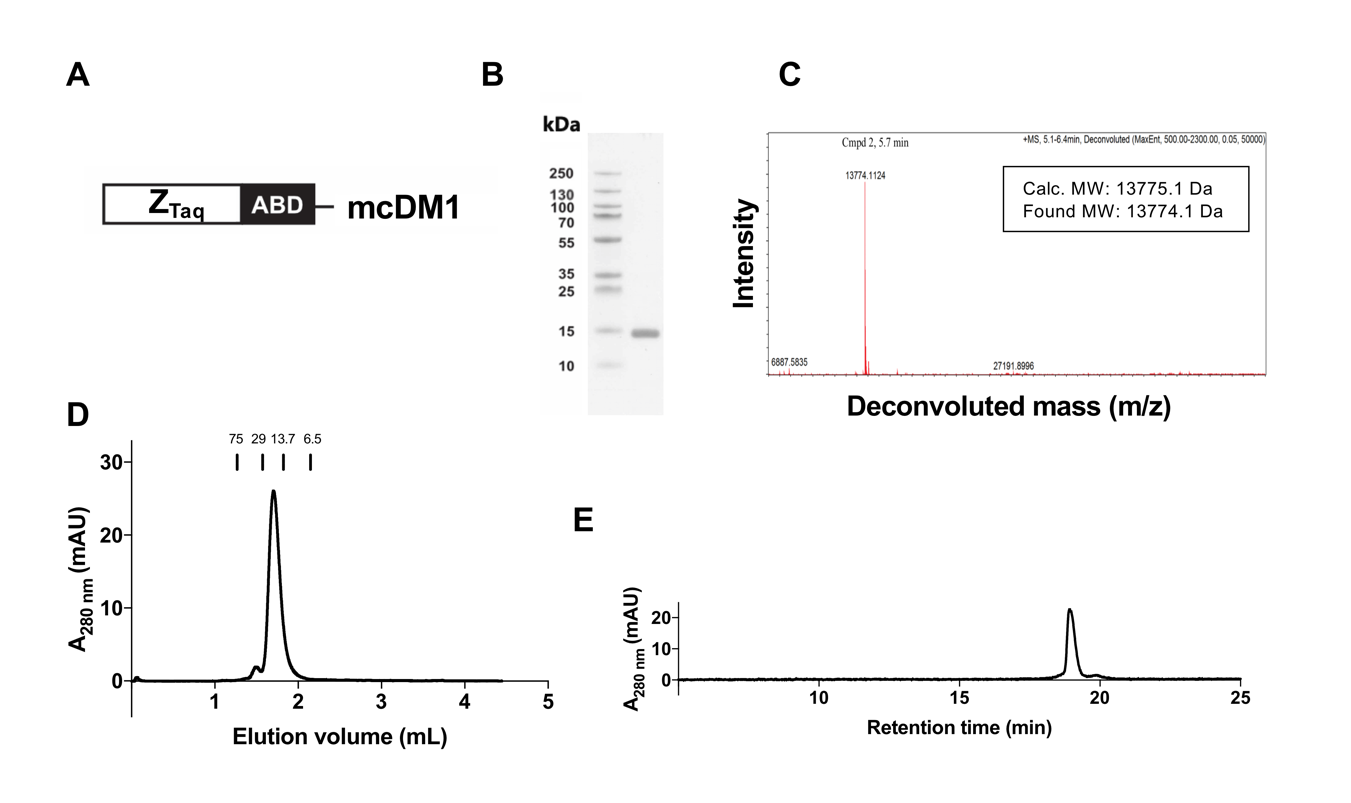
**

**Figure S2.** Characterization of Z_Taq_-ABD-mcDM1. (**A**) Schematic description of the drug conjugate. (**B**) After production and conjugation with mcDM1, Z_Taq_-ABD-mcDM1 was separated by SDS-PAGE under reducing conditions (right lane). The left lane corresponds to separation of marker proteins with molecular weights (in kDa) indicated to the left of the gel. (**C**) The molecular weight of Z_Taq_-ABD-mcDM1 was determined by ESI-TOF mass spectrometry. The drug conjugate was analyzed by size-exclusion chromatographic separation (**D**) and separation by reversed-phage liquid chromatography in a HPLC-setup (**E**).


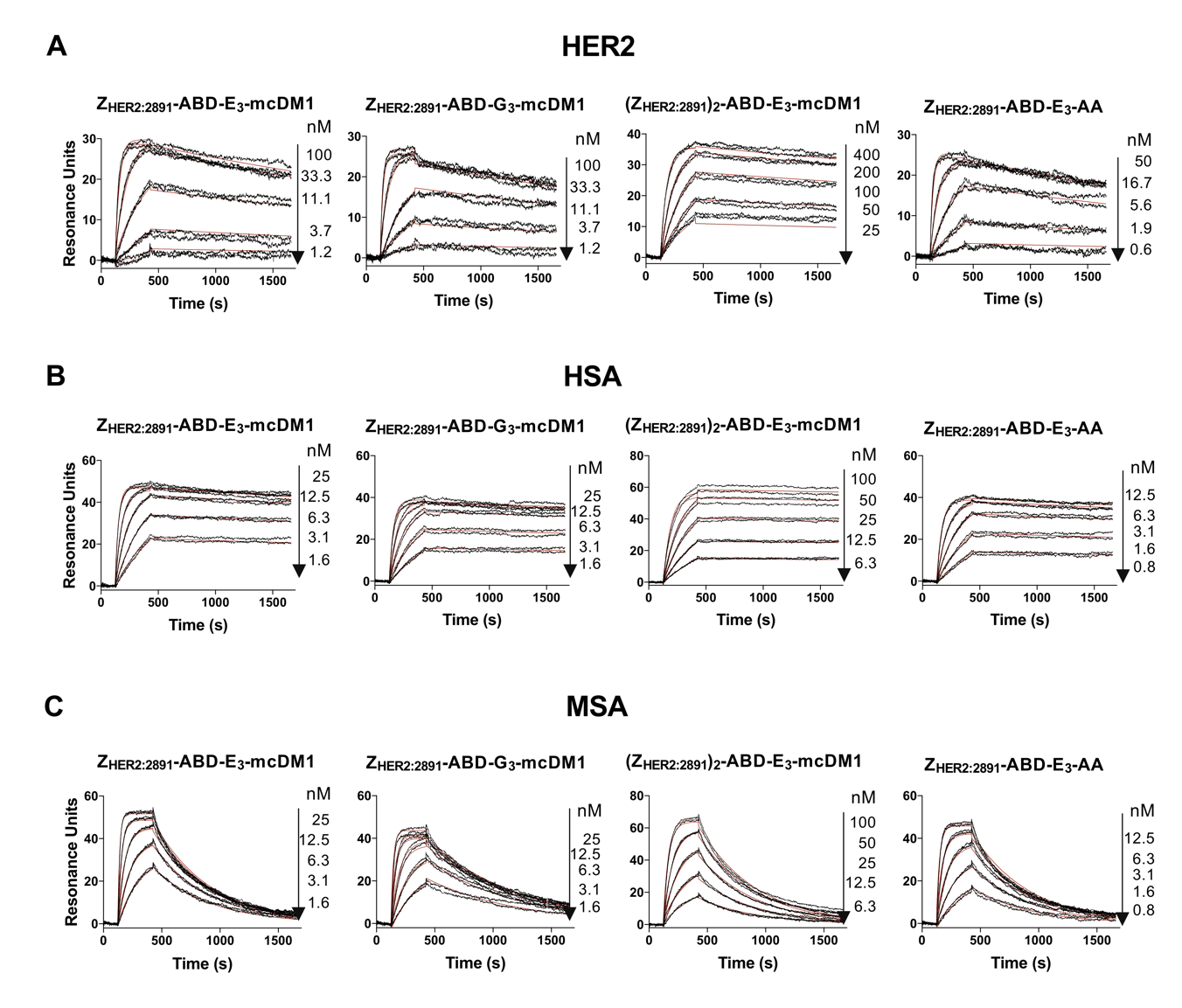


**Figure S3.** Biosensor analysis. Dilution series of the conjugates indicated over each panel were sequentially injected over flow cells with immobilized recombinant HER2 (A), HSA (B), MSA (C), respectively. All experiments were repeated once and each panel is an overlay of all concentrations, in duplicates, for each conjugate. The recorded sensorgrams are shown in black and the data fitted by the biosensor software is in red. The numbers to the right of each panel indicate the concentrations of the injected conjugates (nM) corresponding to each sensorgram.

**Table S1.** Yield, purity and stability after ^99m^Tc-labeling.

|  | **Yield (%)** | **Radiochemical purity (%)** | **Stability under 4 h histidine challenge (%)** | |
| --- | --- | --- | --- | --- |
|  |  |  | Histidine 5000x | Control |
| **^99m^Tc-Z_HER2:2891_-ABD-G_3_-mcDM1** | 95.2 | 99.9 | 98.1 ± 0.0 | 99.2 ± 0.0 |
| **^99m^Tc-Z_HER2:2891_-ABD-E_3_-mcDM1** | 97.5 | 99.8 | 99.1 ± 0.6 | 99.4 ± 0.2 |
| **^99m^Tc-(Z_HER2:2891_)_2_-ABD-E_3_-mcDM1** | 94.1 | 99.9 | 98.9 ± 0.0 | 99.2 ± 0.4 |

**Table S2.** Biodistribution of ^99m^Tc–labelled AffiDCs in tumor bearing mice^a.^

|  | Uptake, %ID/g | | |
| --- | --- | --- | --- |
|  | (Z_HER2:2891_)_2_-ABD-E_3_-mcDM1 | Z_HER2:2891_-ABD-E_3_-mcDM1 | Z_HER2:2891_-ABD-G_3_-mcDM1 |
|  | **4 h** | | |
| Blood | 12 ± 1 | 13 ± 1 | 17.7 ± 0.9 |
| Salivary gland | 3.1 ± 0.6 | 2.5 ± 0.3 | 3.4 ± 0.4 |
| Lung | 5.1 ± 0.5 | 5.5 ± 0.6 | 8 ± 2 |
| Liver | 9 ± 2 * | 6 ± 1 | 9.1 ± 0.8 * |
| Spleen | 6 ± 2 * | 3.5 ± 0.2 | 5.5 ± 0.5 * |
| Small intestine | 3 ± 1 | 2 ± 1 | 3 ± 1 |
| Stomach | 2.0 ± 0.4 | 1.5 ± 0.3 | 2.3 ± 0.4 |
| Large intestine | 2.0 ± 0.1* | 1.5 ± 0.3 | 2.1 ± 0.3 |
| Kidney | 95 ± 8 | 81 ± 12 | 82 ± 8 |
| Tumor | 3.5 ± 0.6 | 3.9 ± 0.5 | 4.2 ± 0.7 |
| Muscle | 0.9 ± 0.09 | 0.9 ± 0.2 | 1.1 ± 0.1 |
| Bone | 1.8 ± 0.5 | 1.6 ± 0.2 | 1.9 ± 0.4 |
|  |  | | |
|  | **24 h** | | |
| Blood | 4.0 ± 0.7 | 6.7 ± 0.3 | 6.8 ± 0.4 |
| Salivary gland | 2.3 ± 0.4 | 2.5 ± 0.4 | 2.3 ± 0.1 |
| Lung | 3.0 ± 1.0 | 3.3 ± 0.3 | 3.5 ± 0.3 |
| Liver | 7.3 ± 1.1 | 5.3 ± 0.4 | 6.2 ± 0.2 |
| Spleen | 4.2 ± 0.8 | 3.5 ± 0.9 | 3.7 ± 0.2 |
| Small intestine | 1.6 ± 0.6 | 1.7 ± 0.7 | 1.9 ± 1.3 |
| Stomach | 1.1 ± 0.1 | 1.17 ± 0.08 | 1.15 ± 0.03 |
| Large intestine | 1.2 ± 0.2 | 1.2 ± 0.1 | 1.3 ± 0.2 |
| Kidney | 59 ± 12 | 56 ± 9 | 46 ± 4 |
| Tumor | 5.7 ± 0.6 | 6.9 ± 0.3 | 6.5 ± 0.2 |
| Muscle | 0.8 ± 0.1 | 1.0 ± 0.2 | 0.8 ± 0.1 |
| Bone | 1.4 ± 0.4 | 1.32 ± 0.07 | 1.2 ± 0.1 |

^a^ The AffiDCs were intravenously injected into female BALB/c-nu/nu mice with SKOV-3 xenografts and biodistribution was determined at 4 and 24 h after injection. The radioactivity is expressed as % of injected dose per gram tissue (%ID/g), and presented as an average value from four animals ± 1 SD. * Significant difference (*p* < 0.05) compared to value for Z_HER2:2891_-ABD-E_3_-mcDM1.
