# Supplementary figures and images for: Drug Conjugates Based on a Monovalent Affibody Targeting Vector Can Efficiently Eradicate HER2 Positive Human Tumors in an Experimental Mouse Model
Source: Cancers (Basel). 2020 Dec 30;13(1):85. doi: 10.3390/cancers13010085 (PMC7794879; doi:10.3390/cancers13010085)

|        |     |       |     |        |      |          |
|--------|-----|-------|-----|--------|------|----------|
| Marker |     | dimar |     | mZTag  | (HE) | Marker   |
| mE3    | MA3 | E3    | mE3 |        | EC1  |          |
| DMI    | DMI | DMI   | IAA | DMI    |      | 20190530 |
|        |     |       |     | Marker |      |          |

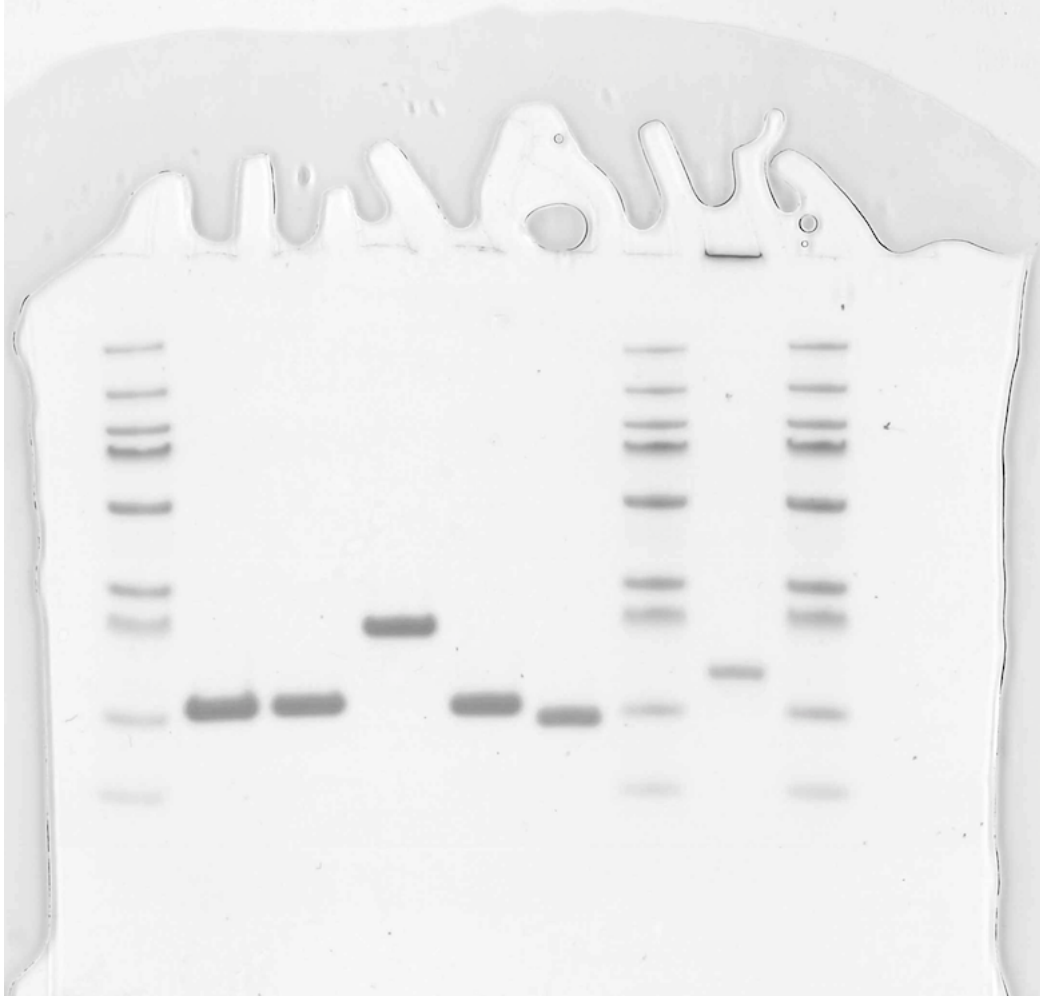

Supplement: Supplementary file 1 [file cancers-13-00085-s001.zip › cancers-1010967-supplementary file-for conversion/cancers-1010967-original-images.pdf]
